# Supplementary material for: Blood flow controls bone vascular function and osteogenesis
Source: Nat Commun. 2016 Dec 6;7:13601. doi: 10.1038/ncomms13601 (PMC5150650; doi:10.1038/ncomms13601)
Supplement: Supplementary Information — Supplementary Figures 1-9 [file ncomms13601-s1.pdf]

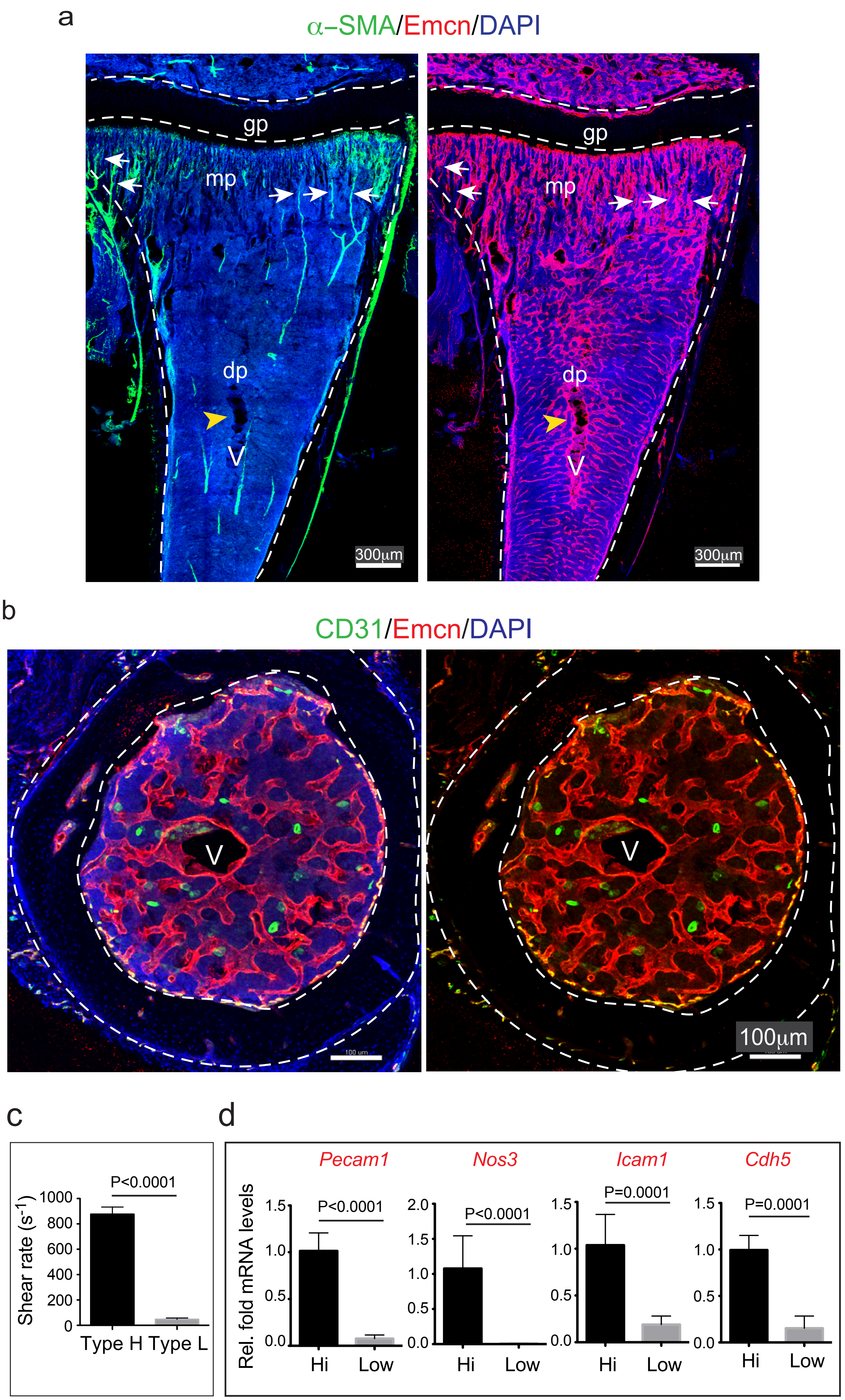
a
α–SMA/Emcn/DAPI
gp
mp
dp
V
300μm
b
CD31/Emcn/DAPI
V
100μm
c
Shear rate (s-1)
P<0.0001
1000
800
600
400
200
0
Type H Type L
d
Pecam1
Nos3
Icam1
Cdh5
Rel. fold mRNA levels
P<0.0001
P<0.0001
P=0.0001
P=0.0001
1.5
1.0
0.5
0.0
2.0
Hi
Low

Supplementary Figure 1. Organization of blood vessels in long bone.

a, Tile scan images of α-SMA (green) and Emcn (red) immunostained blood vessels from 4 week-old tibia. White arrows mark arterioles terminating in the metaphysis (mp). The central large vein (V) in the diaphysis (dp) is indicated by yellow arrowhead. White lines mark growth plate (gp) and borders of compact bone. Nuclei, DAPI (blue).

b, Transverse tibia section immunostained with Emcn (red) and CD31 (green) to differentiate type H, type L and arterial ECs. Dashed lines indicate compact bone. Type H vessels are observed in close proximity to the inner bone surface. Nuclei, DAPI (blue).

c, Graph shows calculated shear rates (s^{-1}) of the endothelium lining type H and type L vessels. Data represent mean±s.d. (n=5 biological replicates). *P* values, two-tailed unpaired t-test.

d, qPCR analysis of *Pecam1*, *Nos3, Icam1* and *Cdh5* expression (normalized to *Actb*) in freshly sorted tibial $CD31^{hi}$ $Emcn^{hi}$ (Hi) ECs relative to $CD31^{lo}$ $Emcn^{lo}$ (Low) ECs. Data represent mean±s.d. (n=5 biological replicates). *P* values, two-tailed unpaired t-test.

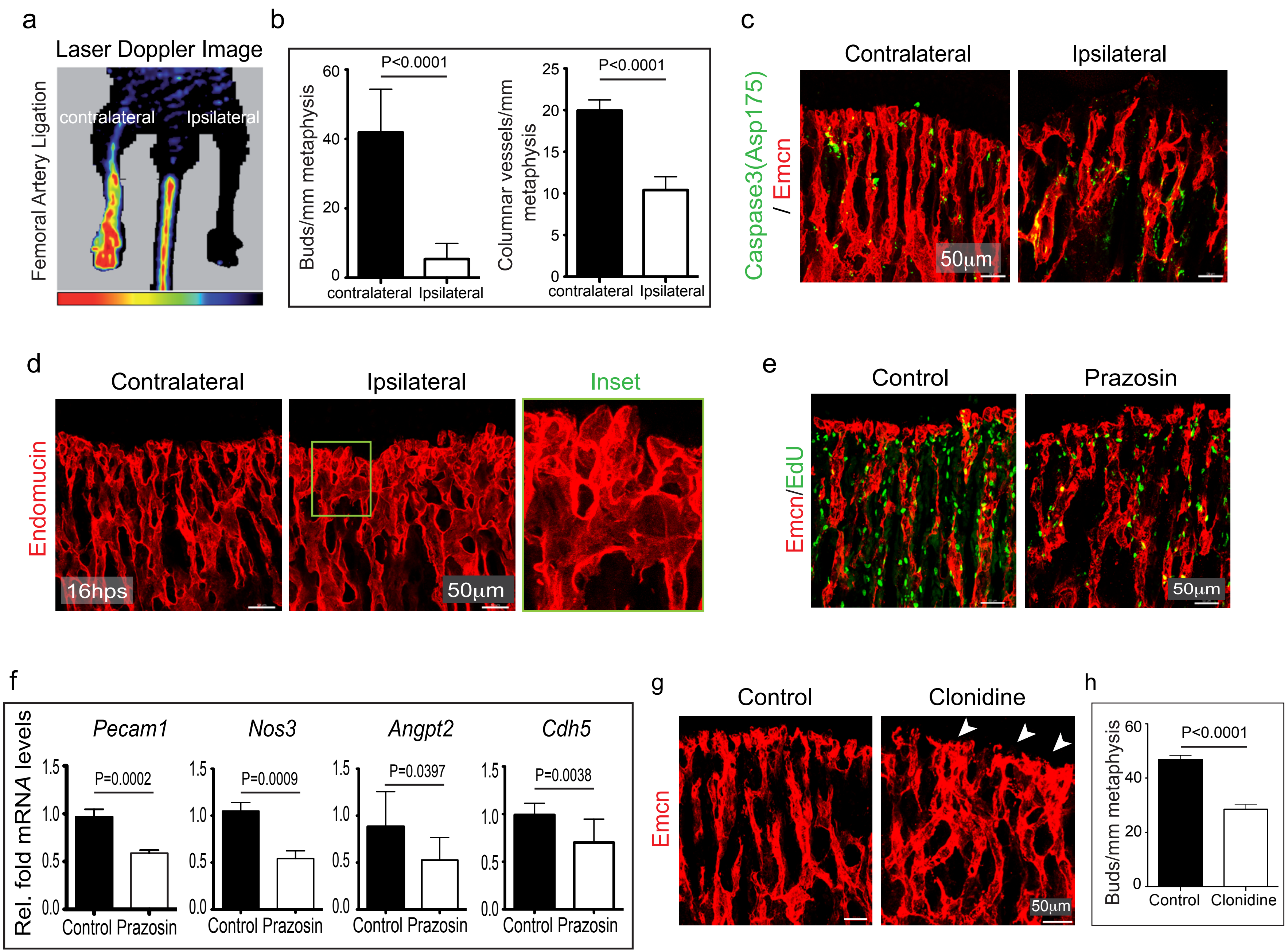
a
Laser Doppler Image
Femoral Artery Ligation
contralateral
Ipsilateral
b
Buds/mm metaphysis
P<0.0001
60
40
20
0
contralateral
Ipsilateral
Columnar vessels/mm metaphysis
P<0.0001
25
20
15
10
5
0
contralateral
Ipsilateral
c
Contralateral
Ipsilateral
Caspase3(Asp175) / Emcn
50μm
d
Contralateral
Ipsilateral
Inset
Endomucin
16hps
50μm
e
Control
Prazosin
Emcn/EdU
50μm
f
Rel. fold mRNA levels
Pecam1
P=0.0002
Nos3
P=0.0009
Angpt2
P=0.0397
Cdh5
P=0.0038
1.5
1.0
0.5
0.0
Control Prazosin
g
Control
Clonidine
Emcn
50μm
h
Buds/mm metaphysis
P<0.0001
60
40
20
0
Control
Clonidine

Supplementary Figure 2. Changes in blood flow affects blood vessels in bone.

a, Laser Doppler imaging of 3 week-old mice after femoral artery ligation shows defective blood flow in the ipsilateral limb. Color intensity indicates perfusion levels.

b, Quantitative analysis of column and bud structures in the vascular front of tibial metaphysis after femoral artery ligation (ipsilateral) compared to unligated (contralateral) limbs. Data represent mean±s.e.m. (n=6 biological replicates). *P* values, two-tailed unpaired t-test.

c, Maximum intensity projections of tibial sections immunostained for cleaved caspase 3 (Asp175; green) and Emcn (red). Apoptosis was comparable in contralateral and ispilateral tibia after femoral artery ligation (48hps).

d, Emcn (red) stained blood vessels after femoral artery ligation (16hps). Increased anastomosis of buds (see higher magnification of inset) on the ligated (ipsilateral) side.

e, Confocal image showing EdU (green) and Endomucin (red) immunostained tibial sections of control and Prazosin-treated tibia. EdU+ cells were decreased after Prazosin treatment.

f, qPCR analysis of sorted total bone ECs showing that Prazosin-treated mice have lower expression of flow-regulated genes such as *Pecam1*, *Nos3*, *Angpt2* and *Cdh5* (normalized to *Actb*) relative to control animals. Data represent mean±s.d. (n=5 biological replicates). P values, two-tailed unpaired t-test.

g, Vascular front structures of control and Clonidine-treated tibiae immunostained for Emcn (red). Note defective formation of buds and columns after Clonidine treatment.

h, Quantification of endothelial bud structures in the vascular front in tibiae shows defective formation of buds after Clonidine treatment relative to control. Data represent mean±s.e.m. (n=5 biological replicates). *P* values, two-tailed unpaired t-test.

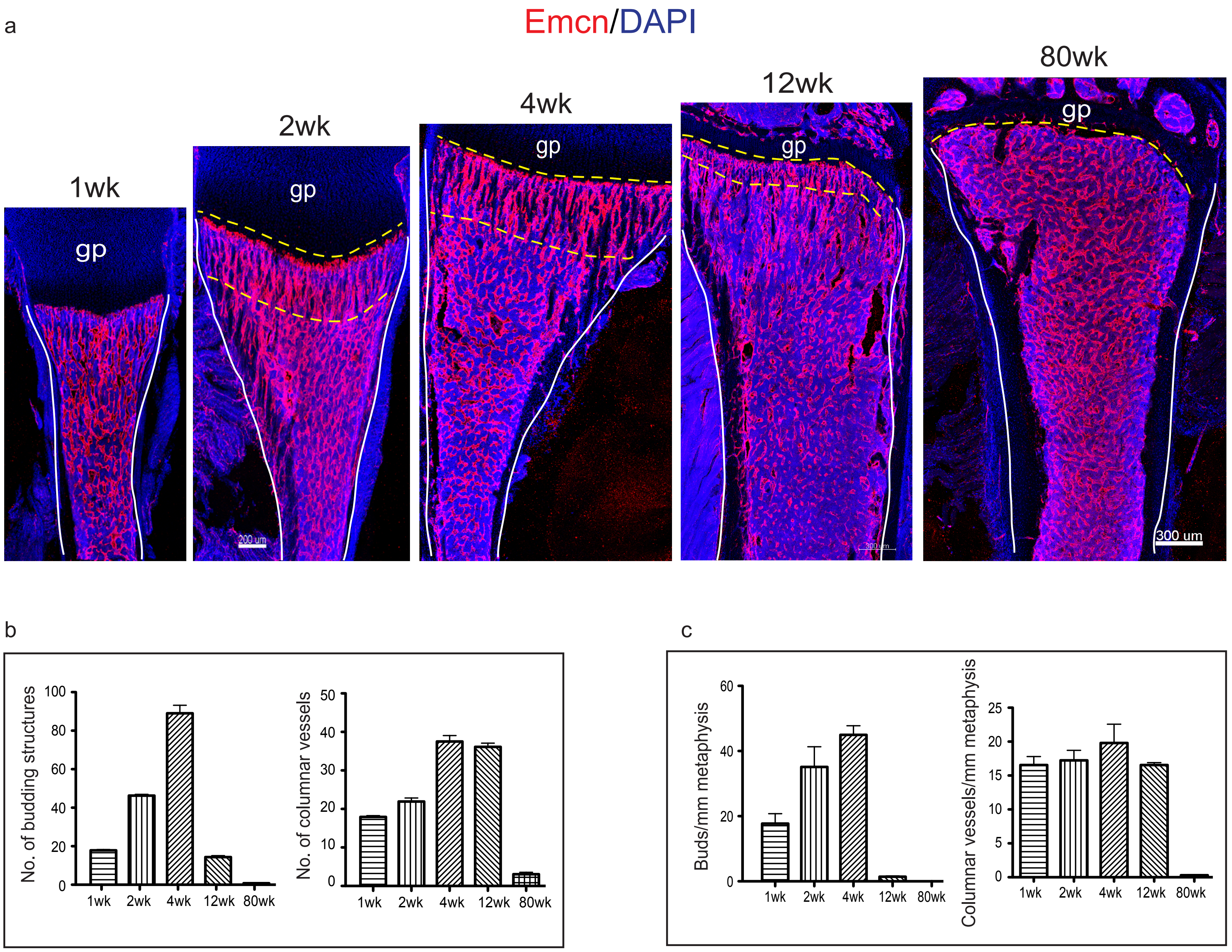

a
Emcn/DAPI
1wk
2wk
4wk
12wk
80wk
gp
gp
gp
gp
gp
200 um
300 um
b
No. of budding structures
100
80
60
40
20
0
1wk
2wk
4wk
12wk
80wk
No. of columnar vessels
50
40
30
20
10
0
1wk
2wk
4wk
12wk
80wk
c
Buds/mm metaphysis
60
40
20
0
1wk
2wk
4wk
12wk
80wk
Columnar vessels/mm metaphysis
25
20
15
10
5
0
1wk
2wk
4wk
12wk
80wk

Supplementary Figure 3. Blood vessel growth in long bone.

a, Tile scans of murine tibia at the indicated ages immunostained for Emcn (red). Note changes in the blood vessel arrangement during growth and ageing. Columnar vessels appear in the metaphysis in postnatal development and are absent in aged mice.

b, **c**, Quantification of vessel buds and columns at the indicated stages. Normalization to the length of the metaphyseal vascular growth front shows that bud abundance reflects changes in postnatal angiogenesis, whereas column number was relatively constant. Data represent mean±s.e.m, n=5 mice in three independent experiments.

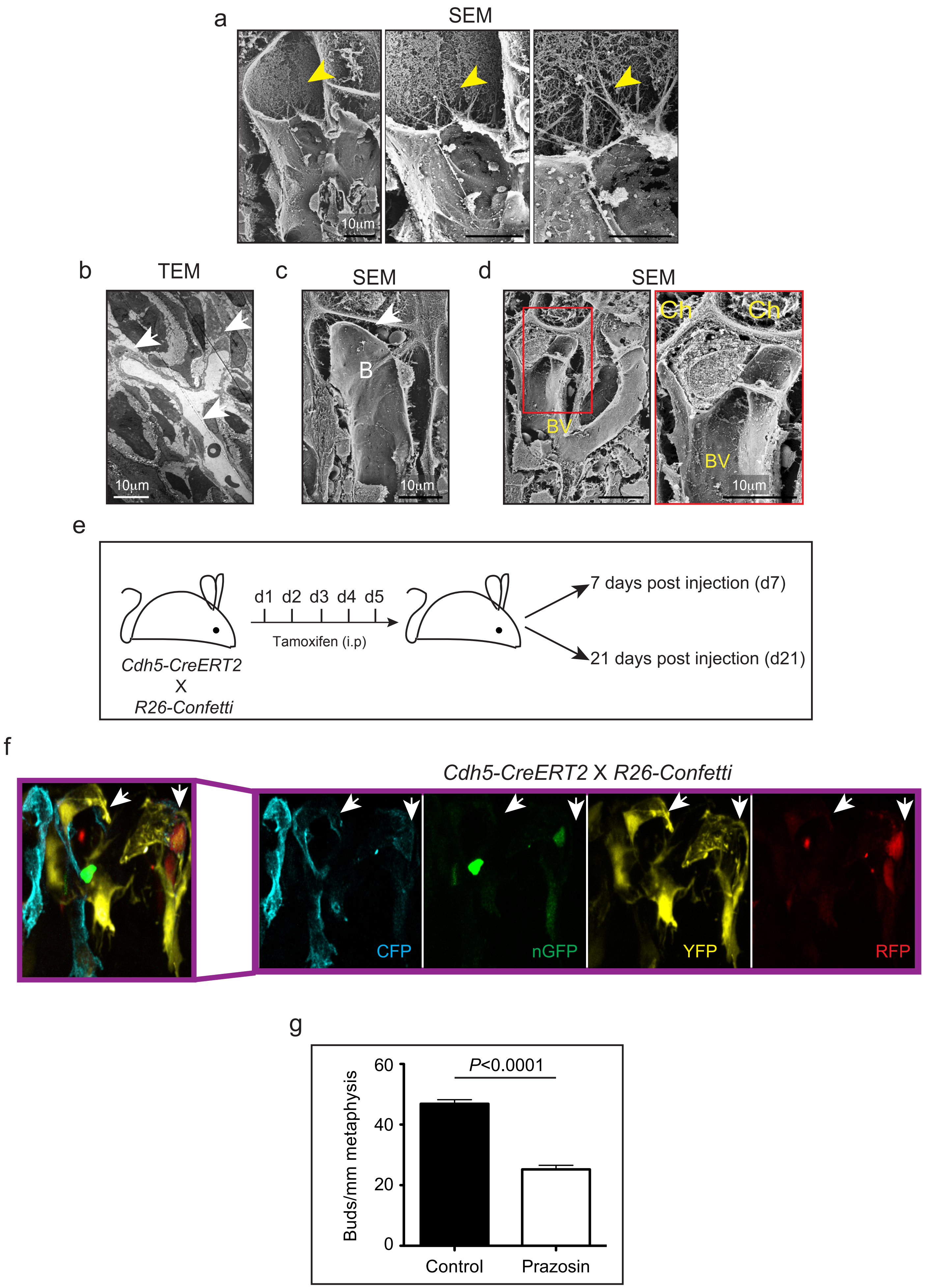

a
SEM
10μm
b
TEM
10μm
c
SEM
B
10μm
d
SEM
BV
Ch
Ch
BV
10μm
e
d1 d2 d3 d4 d5
Tamoxifen (i.p)
7 days post injection (d7)
21 days post injection (d21)
Cdh5-CreERT2
X
R26-Confetti
f
Cdh5-CreERT2 X R26-Confetti
CFP
nGFP
YFP
RFP
g
P<0.0001
Buds/mm metaphysis
60
40
20
0
Control
Prazosin

Supplementary Figure 4. Organization and ultrastructure of vessel buds.

a, Scanning Electron Microscopy (SEM) analysis of vessel front with small filopodia (arrowhead) extending from ECs into the chondrocyte matrix.

b, Transmission Electron Microscopy (TEM) image showing lumen (arrow) in the most distal vessels in 4 week-old metaphysis.

c, **d**, SEM images showing blood vessel (BV) buds (B) lacking filopodia next to intact chondrocytes (Ch).

e, Experimental scheme of tamoxifen-dependent multi-color labeling of ECs in *Cdh5-CreERT2 R26-Confetti* double transgenic mice.

f, Confocal images of 2 week-old *Cdh5-CreERT2 R26-Confetti* tibia isolated 7 days post tamoxifen injection (dpi). Buds (arrows) are composed of multiple ECs.

g, Quantitative analysis of bud structures in Prazosin-treated and control mice. Data represent mean±s.e.m. (n=10 biological replicates). *P* values, two-tailed unpaired t- test.

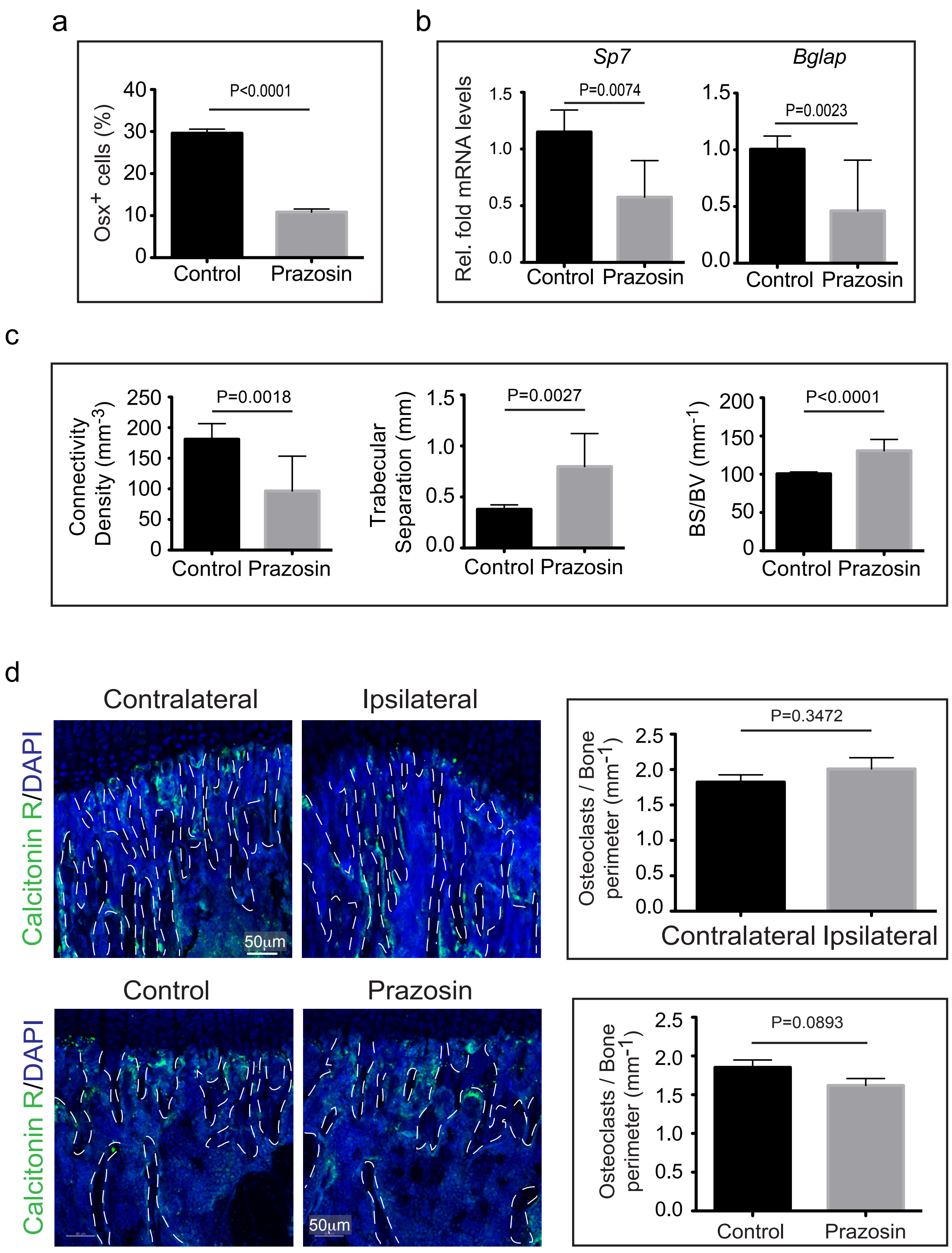
a
Osx+ cells (%)
P<0.0001
Control
Prazosin
b
Sp7
Bglap
Rel. fold mRNA levels
P=0.0074
P=0.0023
Control Prazosin
Control Prazosin
c
Connectivity Density (mm-3)
P=0.0018
Control Prazosin
Trabecular Separation (mm)
P=0.0027
Control Prazosin
BS/BV (mm-1)
P<0.0001
Control Prazosin
d
Contralateral
Ipsilateral
Calcitonin R/DAPI
50μm
Osteoclasts / Bone perimeter (mm-1)
P=0.3472
Contralateral Ipsilateral
Control
Prazosin
Calcitonin R/DAPI
50μm
Osteoclasts / Bone perimeter (mm-1)
P=0.0893
Control
Prazosin

Supplementary Figure 5. Flow-dependent alteration in bone.

a, Quantitation of Osx+ osteoprogenitors in control and Prazosin-treated tibial metaphysis. Data represent mean±s.e.m. (n=5 biological replicates). P value, two-tailed unpaired t-test.

b, qPCR for osteoprogenitor (*Sp7*) and mature osteoblast (*Bglap*) marker gene expression (normalized to *Actb*) in bone lysates from control and Prazosin-treated mice. Data represent mean±s.d. (n=5 biological replicates). P values, two-tailed unpaired *t*-test.

c, Histomorphometrical data derived from micro-CT scans of metaphysis region of tibia. Note that all parameters indicate reduced bone formation after 14 days of Prazosin treatment relative to control. Data represent mean±s.d. (n=5 biological replicates). P values, two-tailed unpaired *t*-test.

d, Osteoclasts in the metaphysis were analyzed by calcitonin receptor (green) immunostaining after femoral artery ligation and Prazosin treatment, as indicated. Nuclei, DAPI (blue). Dashed lines mark trabecular bone. Quantitative data represent mean±s.e.m. (n=6 biological replicates). P value, two-tailed unpaired t-test.

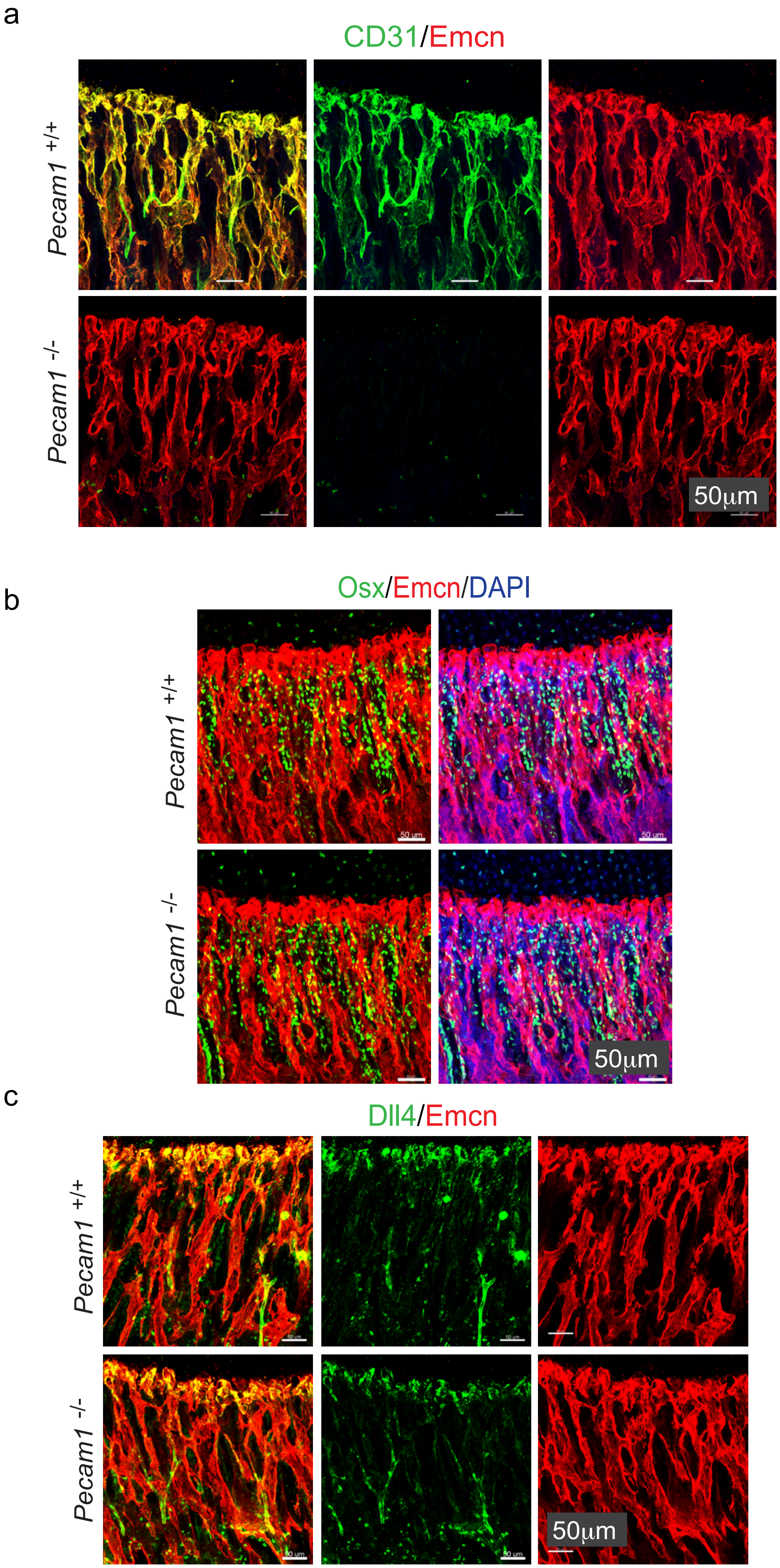
a
CD31/Emcn
Pecam1 +/+
Pecam1 -/-
50μm
b
Osx/Emcn/DAPI
Pecam1 +/+
Pecam1 -/-
50μm
c
Dll4/Emcn
Pecam1 +/+
Pecam1 -/-
50μm

Supplementary Figure 6. Phenotypic analysis of *Pecam1* knockout bone.

a, Maximum intensity projections of mouse tibia immunostained for CD31 (green) and Emcn (red) confirming the absence of Pecam1 expression in *Pecam1* knockout ($Pecam1^{-/-}$) mice compared to their littermate controls (*Pecam1*$^{+/+}$). No significant change was observed in the $Pecam1^{-/-}$ vasculature.

b, Confocal images showing tibial sections immunostained for Emcn (red) and Osx (green). Pecam1 deletion (*Pecam1*$^{-/-}$) did not affect osteoprogenitors in bone.

c, Confocal images show comparable Dll4 expression (green) in 4 week-old $Pecam1^{-/-}$ and control tibia.

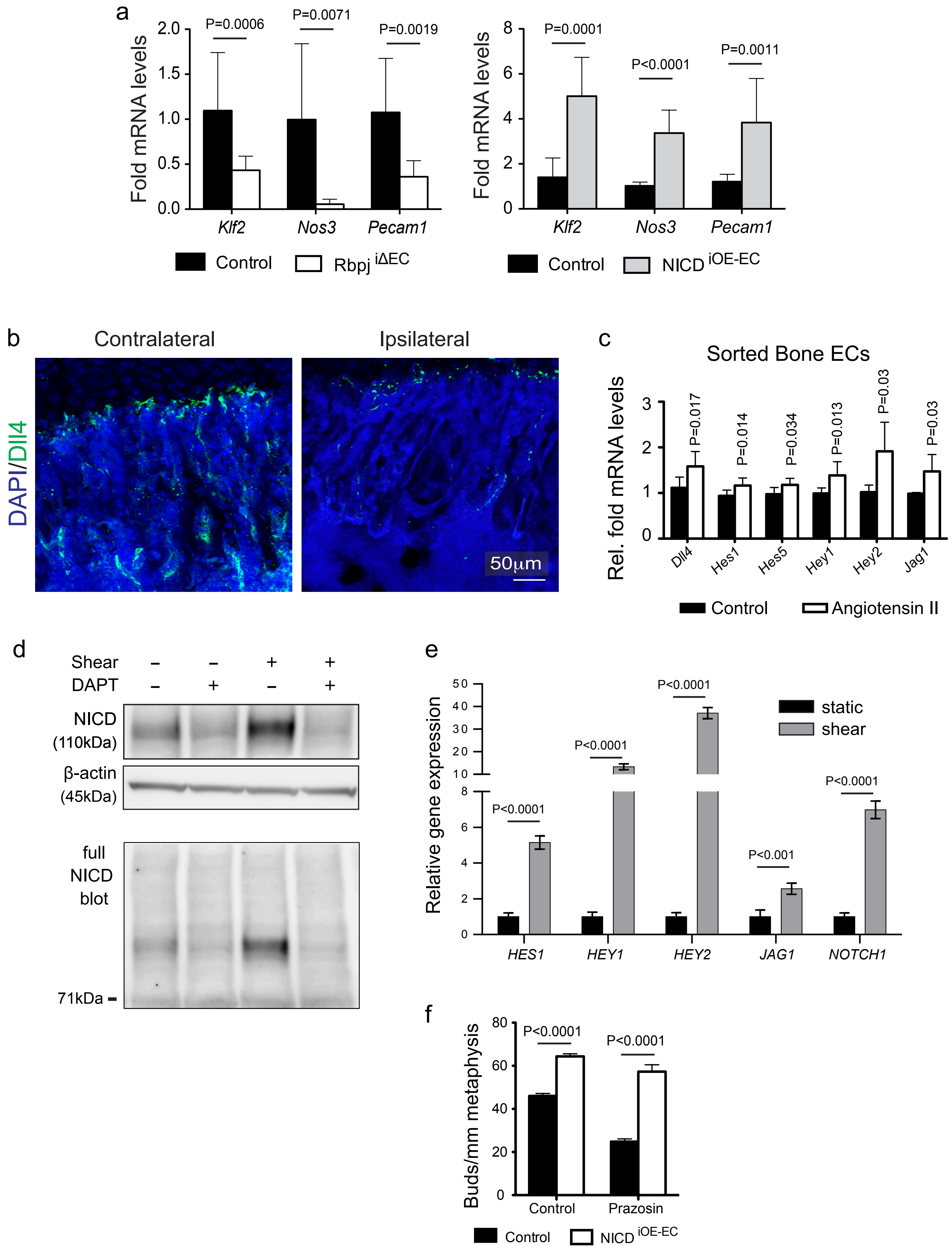
a
Fold mRNA levels
2.0
1.5
1.0
0.5
0.0
P=0.0006
P=0.0071
P=0.0019
Klf2
Nos3
Pecam1
Control
Rbpj iΔEC
Fold mRNA levels
8
6
4
2
0
P=0.0001
P<0.0001
P=0.0011
Klf2
Nos3
Pecam1
Control
NICD iOE-EC
b
Contralateral
Ipsilateral
DAPI/Dll4
50μm
c
Sorted Bone ECs
Rel. fold mRNA levels
3
2
1
0
P=0.017
P=0.014
P=0.034
P=0.013
P=0.03
P=0.03
Dll4
Hes1
Hes5
Hey1
Hey2
Jag1
Control
Angiotensin II
d
Shear – – + +
DAPT – + – +
NICD
(110kDa)
β-actin
(45kDa)
full
NICD
blot
71kDa
e
Relative gene expression
50
40
30
20
10
8
6
4
2
0
P<0.0001
P<0.0001
P<0.0001
P<0.001
P<0.0001
static
shear
HES1
HEY1
HEY2
JAG1
NOTCH1
f
Buds/mm metaphysis
80
60
40
20
0
P<0.0001
P<0.0001
Control
Prazosin
Control
NICD iOE-EC

Supplementary Figure 7. Changes in bone endothelium upon flow modulation.

a, Graph shows qPCR analysis of flow downstream target genes such as *Klf2*, *Nos3*, *Pecam1* in sorted bone endothelial cells of EC-specific Notch mutants. Levels of these transcripts were significantly reduced in $Rbpj^{i\Delta EC}$ bone ECs and higher in $NICD^{iOE\text{-}EC}$ bone ECs relative to littermate control cells. Data represent mean±s.d. (n=7 biological replicates). P values, two-tailed unpaired t-test.

b, Tibial sections of contralateral and ipsilateral limbs. Femoral artery ligation led to decreased Dll4 (green) expression. Nuclei, DAPI (blue).

c, qPCR analysis of *Dll4*, *Hes1, Hes5, Hey1, Hey2* and *Jag1* expression in ECs sorted from control and angiotensin II-treated long bones. Data represent mean±s.d. (n=5 biological replicates). P values, two-tailed unpaired t-test.

d, Western blot showing Notch activation (NICD) in cultured human umbilical vein ECs (HUVECs) exposed to laminar flow (15 dyn/cm^2) for 4 hours. Actin is shown as loading control. Molecular weight marker (kD) is indicated.

e, RT-qPCR analysis of Notch target gene expression in cultured HUVECs transcript exposed to laminar flow 15 dyn/cm^2) for 4 hours. Data represent mean±s.d. P values, one-way ANOVA-Dunnet's post hoc test.

f, Quantitation of bud density in vehicle (control) and Prazosin-treated $NICD^{iOE\text{-}EC}$ transgenics and littermate controls. Data represent mean±s.e.m. (n=4 biological replicates). *P* values, two-tailed unpaired t-test.

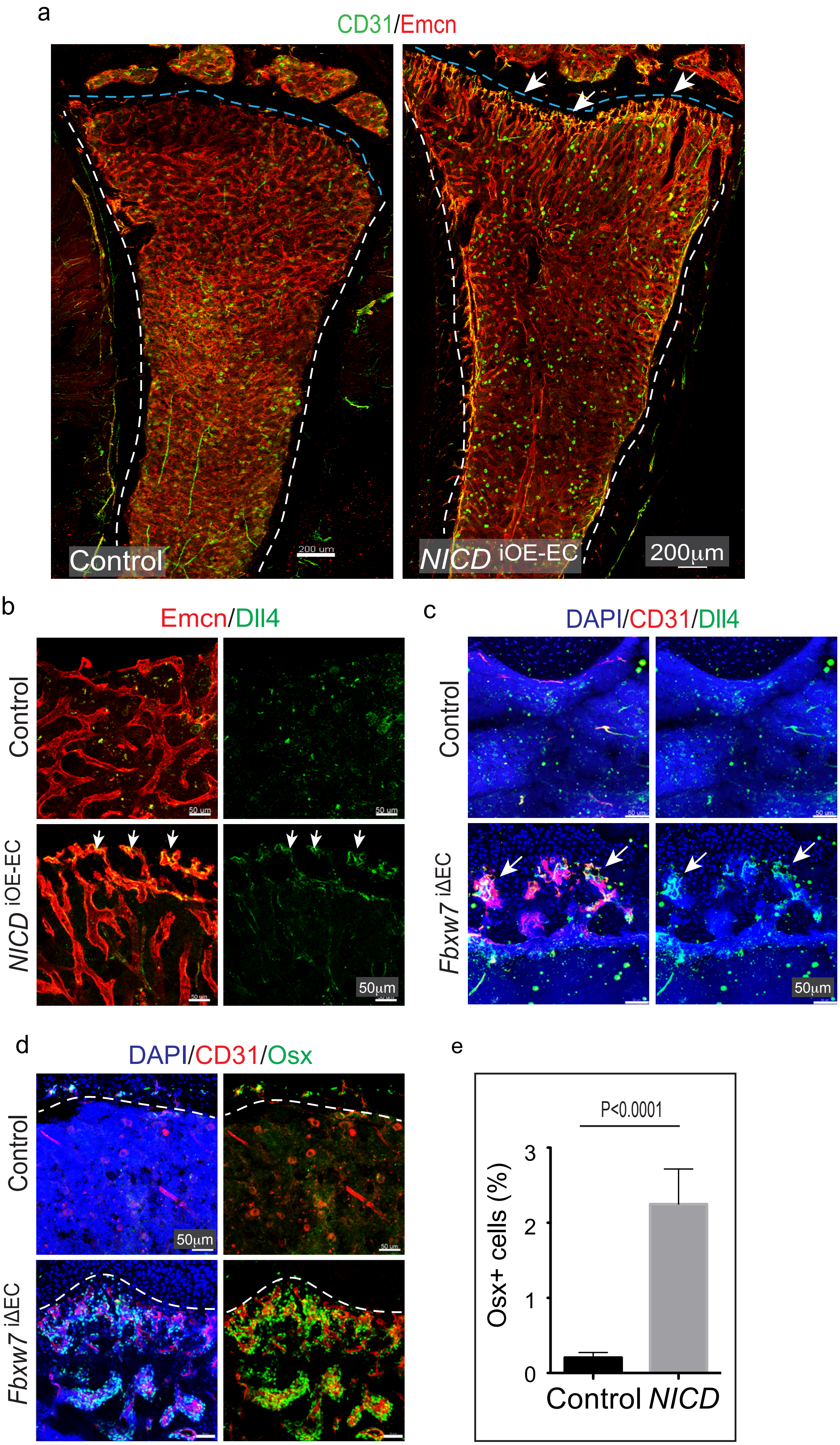

a
CD31/Emcn
Control
200 um
NICD iOE-EC
200µm
b
Emcn/Dll4
Control
NICD iOE-EC
50µm
c
DAPI/CD31/Dll4
Control
Fbxw7 iΔEC
50µm
d
DAPI/CD31/Osx
Control
50µm
Fbxw7 iΔEC
e
P<0.0001
Osx+ cells (%)
3
2
1
0
Control
NICD

Supplementary Figure 8. Notch signaling in aged bone endothelium.

a, Tile scan confocal images of aged *NICD*$^{iOE\text{-}EC}$ and littermate control tibiae immunostained for CD31 (green) and Emcn (red). Note increase in CD31+ vessels and buds (arrows) in *NICD*$^{iOE\text{-}EC}$ bone near chondrocyte region (dashed blue line).

b, Maximum intensity projections of aged (70 week-old) tibia immunostained for Dll4 (green). Note low Dll4 levels in control Emcn+ (red) vessels and increased expression and bud formation (arrows) in EC-specific Notch gain-of-function (*NICD* $^{iOE\text{-}EC}$) mice.

c, Maximum intensity projections of aged control and *Fbxw7*$^{i\Delta EC}$ tibiae immunostained for Dll4 (green). EC-specific inactivation of *Fbxw7* led to emergence of new CD31+ (red) and Dll4+ buds (arrows). Nuclei, DAPI (blue).

d, Osterix+ (Osx, green) osteoprogenitors and CD31+ (red) vessels were strongly increased in aged *Fbxw7*$^{i\Delta EC}$ mutants relative to control littermates. Nuclei, DAPI (blue).

e, Quantitation of Osx+ cells in the metaphyseal region of aged *NICD*$^{iOE\text{-}EC}$ and control tibiae. Data represent mean±s.e.m, n=4 mice in 3 independent experiments. *P* values, two-tailed unpaired t-test.

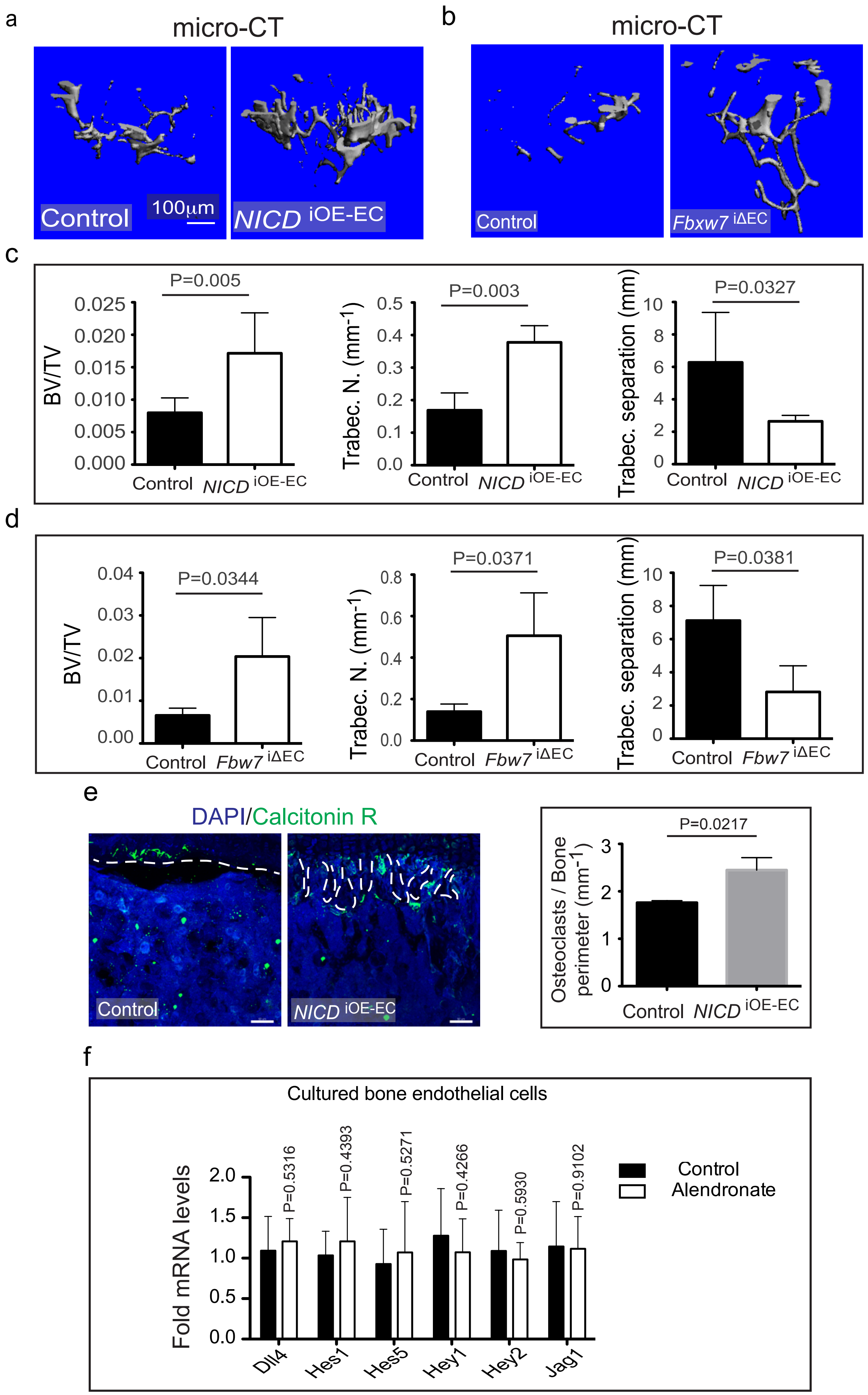

a
micro-CT
Control
100μm
NICD iOE-EC
b
micro-CT
Control
Fbxw7 iΔEC
c
P=0.005
BV/TV
0.025
0.020
0.015
0.010
0.005
0.000
Control NICD iOE-EC
P=0.003
Trabec. N. (mm-1)
0.5
0.4
0.3
0.2
0.1
0.0
Control NICD iOE-EC
P=0.0327
Trabec. separation (mm)
10
8
6
4
2
0
Control NICD iOE-EC
d
P=0.0344
BV/TV
0.04
0.03
0.02
0.01
0.00
Control Fbw7 iΔEC
P=0.0371
Trabec. N. (mm-1)
0.8
0.6
0.4
0.2
0.0
Control Fbw7 iΔEC
P=0.0381
Trabec. separation (mm)
10
8
6
4
2
0
Control Fbw7 iΔEC
e
DAPI/Calcitonin R
Control
NICD iOE-EC
P=0.0217
Osteoclasts / Bone perimeter (mm-1)
3
2
1
0
Control NICD iOE-EC
f
Cultured bone endothelial cells
Fold mRNA levels
2.0
1.5
1.0
0.5
0.0
P=0.5316
P=0.4393
P=0.5271
P=0.4266
P=0.5930
P=0.9102
Dll4
Hes1
Hes5
Hey1
Hey2
Jag1
Control
Alendronate

Supplementary Figure 9. Histomorphometric analysis of aged long bone after Notch activation.

a, Representative micro-CT images of mineralized regions in aged *NICD*$^{iOE\text{-}EC}$ and control tibial metaphysis.

b, Three dimensional rendering of micro-CT scans performed on tibia from aged *Fbxw7*$^{i\Delta EC}$ mutants and control littermates.

c, **d**, Histomorphometric parameters showing improved bone formation in *Fbxw7*$^{i\Delta EC}$ (**c**) and *NICD*$^{iOE\text{-}EC}$ (**d**) long bone relative to littermate controls. Data represent mean±s.d. (n=4 biological replicates for each group). P value, two-tailed unpaired t-test.

e, Osteoclasts (calcitonin receptor, green) in aged *NICD*$^{iOE\text{-}EC}$ and control tibiae. Nuclei, DAPI (green). Graph shows increased osteoclast number in aged *NICD*$^{iOE\text{-}EC}$ mutants. Data represent mean±s.e.m. (n=4 biological replicates). P value, two-tailed unpaired t-test.

f, Cultured bone endothelial cells were analyzed for the expression of Notch target genes after Alendronate treatment *in vitro*. No significant changes in the expression of Notch target genes were observed after an 8 hour treatment of Alendronate suggesting that the bisphosphonate does not influence endothelial Notch signaling directly. Data represent mean±s.d. (n=5 biological replicates). P value, two-tailed unpaired t-test.
